# Integrated Metabolomic and Transcriptomic Analysis of Modified Nucleosides for Biomarker Discovery in Clear Cell Renal Cell Carcinoma

**DOI:** 10.3390/cells12081102

**Published:** 2023-04-07

**Authors:** Daniel A. Mohl, Simon Lagies, Kyra Zodel, Matthias Zumkeller, Asin Peighambari, Athina Ganner, Dietmar A. Plattner, Elke Neumann-Haefelin, Mojca Adlesic, Ian J. Frew, Bernd Kammerer

**Affiliations:** 1 Core Competence Metabolomics, Hilde-Mangold-Haus, University of Freiburg, 79104 Freiburg, Germany; daniel.mohl@ocbc.uni-freiburg.de (D.A.M.);; 2 Institute of Organic Chemistry, University of Freiburg, 79104 Freiburg, Germany; 3 Institute of Medical Microbiology and Hygiene, Faculty of Medicine, Medical Center—University of Freiburg, 79104 Freiburg, Germany; 4 Department of Internal Medicine I, Hematology, Oncology and Stem Cell Transplantation, Faculty of Medicine, Medical Centre—University of Freiburg, 79106 Freiburg, Germany; 5 Renal Division, Department of Medicine, Medical Center—University of Freiburg, Faculty of Medicine, University of Freiburg, 79106 Freiburg, Germany; 6 German Cancer Consortium (DKTK), Partner Site Freiburg, and German Cancer Research Center (DKFZ), 69120 Heidelberg, Germany; 7 Signalling Research Centre BIOSS, University of Freiburg, 79104 Freiburg, Germany; 8 Comprehensive Cancer Center Freiburg (CCCF), Faculty of Medicine and Medical Center—University of Freiburg, 79106 Freiburg, Germany; 9 Spemann Graduate School of Biology and Medicine (SGBM), University of Freiburg, 79104 Freiburg, Germany

**Keywords:** biomarkers, biomarker discovery, ccRCC, kidney cancer, metabolomics, mass spectrometry, HPLC, modified nucleosides, RNA-seq, transcriptomics

## Abstract

Clear cell renal cell carcinoma (ccRCC) accounts for ~75% of kidney cancers. The biallelic inactivation of the von Hippel–Lindau tumor suppressor gene (*VHL*) is the truncal driver mutation of most cases of ccRCC. Cancer cells are metabolically reprogrammed and excrete modified nucleosides in larger amounts due to their increased RNA turnover. Modified nucleosides occur in RNAs and cannot be recycled by salvage pathways. Their potential as biomarkers has been demonstrated for breast or pancreatic cancer. To assess their suitability as biomarkers in ccRCC, we used an established murine ccRCC model, harboring *Vhl*, *Trp53* and *Rb1* (VPR) knockouts. Cell culture media of this ccRCC model and primary murine proximal tubular epithelial cells (PECs) were investigated by HPLC coupled to triple-quadrupole mass spectrometry using multiple-reaction monitoring. VPR cell lines were significantly distinguishable from PEC cell lines and excreted higher amounts of modified nucleosides such as pseudouridine, 5-methylcytidine or 2′-O-methylcytidine. The method’s reliability was confirmed in serum-starved VPR cells. RNA-sequencing revealed the upregulation of specific enzymes responsible for the formation of those modified nucleosides in the ccRCC model. These enzymes included Nsun2, Nsun5, Pus1, Pus7, Naf1 and Fbl. In this study, we identified potential biomarkers for ccRCC for validation in clinical trials.

## 1. Introduction

In 2020, kidney cancer was diagnosed in more than 400,000 people worldwide [1]. Over 90% of all primary renal neoplasms originate from renal cell carcinomas (RCCs). This heterogeneous cancer, which derives mainly from renal tubular epithelial cells, has more than 10 histological and molecular subtypes and is among the ten most common cancers worldwide. Clear cell renal cell carcinoma (ccRCC) is the most common type of kidney cancer, accounting for 75% of all kidney tumors and causing the most kidney-cancer-related deaths. Approximately 30% of patients with localized disease will develop metastasis despite nephrectomy, causing high mortality and requiring systemic therapies [2,3].

In the majority of ccRCC cases, the biallelic loss of the von Hippel–Lindau (*VHL*) tumor suppressor gene is the main truncal oncogenic driving event [3,4]. The long latency of the development of ccRCC in individuals carrying germline *VHL* mutations and the absence of tumors in murine models with *Vhl* knockouts show that the *VHL* loss alone is insufficient to cause ccRCC. Other genetic or epigenetic events cooperate with the loss of VHL function to cause tumor formation [2]. Aberrations and mutations in cell cycle regulatory genes such as *TP53*, *CDKN2A* and *MYC* as well as *PI3K* regulatory genes such as *PIK3CA*, *PTEN*, *MTOR* and *TSC1* or epigenetic regulatory genes such as *PBRM1*, *BAP1*, *SETD2* and *KDM5C* are recurrently altered in ccRCC and are believed to promote tumor evolution and growth [5,6]. VHL is the protein for the substrate recognition of an E3 ligase complex that ubiquitinates HIF-1α and HIF-2α, which causes the proteasome-mediated degradation of HIF [7,8,9]. The inactivation of VHL causes the aberrant activation of HIF target genes that regulate many cellular processes including glycolysis, apoptosis and angiogenesis. Therefore, ccRCC tumors are strongly vascularized and rich in lipids and glycogen [10,11]. ccRCC is often diagnosed incidentally when patients undergo abdominal imaging techniques such as ultrasonography or CT scans. Patients might also be diagnosed with ccRCC when presenting with gross haematuria, palpable abdominal mass or flank pain. When diagnosed incidentally or after symptoms occur, it might be too late for successful therapy and a good outcome [2]. Hence, the discovery of diagnostic biomarkers for early detection is crucial.

It has been shown that ccRCC exhibits distinct molecular alterations on the genetic, epigenetic, transcriptomic, proteomic and metabolomic levels that potentially could be exploited as biomarkers [11]. Biomarkers are measurable indicators for disease diagnosis, prognosis, therapy surveillance, and outcome. They can be single molecules, genes, proteins, or signatures of these. Molecular biomarkers can be obtained from body fluids such as serum, plasma or urine. Non-invasive methods, such as urine samples, are preferred. An ideal biomarker should be accurate, reliable, specific, cost-effective and easily measurable. On the metabolomics level, modified ribonucleosides (hereinafter referred to as modified nucleosides) have been shown to have potential as biomarkers in breast or pancreatic cancer [12,13,14]. Nucleosides comprise a ribose moiety coupled to a nucleobase via a glyosidic bond. Nucleosides are components of all different types of RNA such as tRNA, mRNA, rRNA and snRNA [15]. These RNAs are made up of the basic nucleosides adenosine, guanosine, uridine and cytidine, which can be modified post-transcriptionally by different enzymes causing methylations, hydroxylations, reductions, sulfur/oxygen substitutions or the addition of diverse sidechains [16]. More than 150 modified nucleosides are known, which occur in different types of RNA [17]. By far the most modifications can be found in tRNAs in terms of the extent and modification diversity [18]. These modifications take place in the nucleus in the pre-RNA and are mostly introduced post-transcriptionally by highly specific enzymes to the common nucleosides adenosine, guanosine, cytidine and uridine [18]. The second most common modifications occur in rRNA where ribose-methylated residues are prevalent [19]. Common nucleosides can be recycled by salvage pathways. Initially, the RNAs are degraded to oligonucleotides by endonucleases, followed by the cleavage of phosphatases to nucleosides, which can be phosphorylated again to build up nucleotides such as ATP, GTP, UTP or CTP or cleaved into ribose-1-phosphate and the respective nucleobase by special phosphorylases. The sugar phosphates and the bases can be also excreted from the cell and metabolized to CO_2_, NH_3_, uric acid, β–aminoisobutyrate or β-alanine. However, modified nucleosides cannot be salvaged by these pathways due to the lack of specific phosphorylases for modified nucleosides and thus are excreted by the cells, ultimately accumulating quantitatively in the urine [12]. Since cancer cells have an altered RNA metabolism and an increased turnover of RNAs in contrast to normal cells, modified nucleosides are excreted in larger amounts, which makes them interesting as biomarkers [20,21,22].

High-performance liquid chromatography coupled to different types of mass spectrometers (HPLC-MS) represents a powerful analytical technique due to its high sensitivity, selectivity and accuracy. This enables the identification and quantification of specific biomarker molecules, even in complex biological matrices, leading to a wide range of applications in clinical diagnostics, drug development and biomarker discovery. Excreted nucleosides from (cancer) cells need to be qualified and quantified to assess their suitability as biomarkers. In the past, nucleosides from different cancers such as breast or pancreatic cancer have been measured with LC ion trap MS, MALDI-TOF-MS or HPLC coupled to triple-quadrupole (QqQ) MS or TOF-MS using electrospray ionization (ESI) [13,21,22,23,24]. For that reason, this study uses a reversed phase HPLC coupled to QqQ-MS with ESI. Murine ccRCC cell lines were analyzed together with murine proximal tubular epithelial cells as controls, since ccRCC originates from the proximal tubules. This murine model has the tubule-specific deletion of *Vhl*, *Trp53*, and *Rb1*, abbreviated as VPR, which is described as an accurate model of ccRCC and shows mutational, transcriptional, proteomic, histological and immunohistochemical similarities to human ccRCC [9,25].

The aim of this work was to determine whether there are any metabolic differences between normal (PEC) and cancer cells (VPR) regarding modified nucleosides and to discover potential candidates for novel biomarkers for ccRCC. 

## 2. Materials and Methods

### 2.1. Cell Culture and Sample Preparation

Cells acquired from VPR tumors were isolated as previously described [26]. In brief, tumor tissue was cut and incubated with collagenase II. The suspension was filtered through a 70 µm cell strainer and grown in K1 medium (Dulbecco’s Modified Eagle Medium/Nutrient Mixture F12-Ham, Sigma-Aldrich (now Merck, Darmstadt, Germany), with added Pen-Strep (2% *v*/*v*), L-glutamine (1% *w*/*v*), insulin (5 μg/mL), prostaglandin E1 (1.25 ng/mL), triiodothyronine (34 pg/mL), Apo-transferrin (5 μg/mL), sodium selenite (1.73 ng/mL), hydrocortisone (18 ng/mL) and epidermal growth factor (25 ng/mL)) with 10% fetal calf serum (FCS) (*v*/*v*, Gold, FCS) at 5% CO_2_ and 37 °C in an incubator (Heracell 240i), split every 3–4 days and not passaged more than 20 times. Primary renal epithelial cells (PECs) were isolated from mouse kidneys as described in [26] and grown in the same medium as VPR cell lines. The starved VPR cells were cultivated in K1 as above, but the medium contained only 0.5% FCS. The cell-conditioned culture media were centrifuged, and the supernatant media, containing all the exometabolites were aliquoted, were snap frozen in liquid nitrogen and stored at −80 °C. In total, 100 µL of the thawed supernatants were pooled with 900 µL of cold precipitation solution (3:1, acetonitril:methanol, with 1 µg/mL isoguanosine as internal standard) to precipitate all proteins. After vortexing and centrifuging (45 min, 20,000× *g*, 4 °C), the supernatants were evaporated in the vacuum concentrator, and the pellets were reconstituted in 100 µL of ddH_2_O. A total of 70 µL was transferred in an LC-vial, and 20 µL of each sample was pooled to create a mixed quality control sample. 

### 2.2. LC-MS

The nucleosides were separated by reversed phase HPLC (Waters Acquity HSS T3, Waters GmbH, Eschborn, Germany; Agilent LC 1290 Infinity, Agilent Technologies, Waldbronn, Germany) coupled to a triple-quadrupole mass spectrometer (Agilent Technologies 6460 Triple Quad LC/MS). As solvents, ddH_2_O (A) and methanol with 0.1% formic acid (B) were used. The chromatography program was 100% A for 3 min, to 72% A within 2 min, to 65% A within 3 min, to 2% A within 1 min, held for 4 min, to 100% A within 0.5 min and held for 6.5 min. The flow rate was set to 300 µL/min with the column temperature set to 50 °C. For the nucleosides, a targeted LC-MS MRM (multiple-reaction-monitoring) and NLS (neutral loss) analysis was carried out. The MRM method previously described by Schlimpert et al. was slightly modified and used [27]. For the MS analysis, the following parameters were applied: the gas temperature was set to 300 °C with a flow rate of 7 L/min. The sheath gas flow rate was 7 L/min at 350 °C. The Nebulizer pressure was 50 psi. The mass spectrometer was operated with +4 kV and 500 V nozzle voltage. Details about MRM transitions can be found in [12]. The samples were kept at 4 °C, and 10 µL was injected in a randomized order to eliminate temporal aberrations. Quality control samples were injected regularly in between. Table 1 depicts the nucleosides identified in this study with their corresponding abbreviations.

### 2.3. Data Processing and Statistical Analysis

Agilent MassHunter Qualitative Analysis and Agilent MassHunter Quantitative Analysis were used for post processing of the LC-MS data. The nucleosides’ peak intensities were normalized to the internal standard isoguanosine and phenol red, the pH indicator of the cell culture medium, which was present in all samples in equal amounts.

For statistical analysis, Microsoft Excel 2016 and MetaboAnalyst 5.0 were used [28]. The generated data were normalized in MetaboAnalyst using range scaling, which has the advantage of all metabolites becoming equally important so that they can be compared relative to their response range [29]. Hierarchical clustering was achieved with MetaboAnalyst using the normalized peak areas with Euclidian distance measurement and Ward’s minimum variance. For the analysis of variance (ANOVA), a *q*-value of 0.05 was used, which was obtained by multiple testing correction (FDR).

## 3. Results and Discussion

### 3.1. Analysis under 10% Serum Growth Conditions

**Figure 1:**
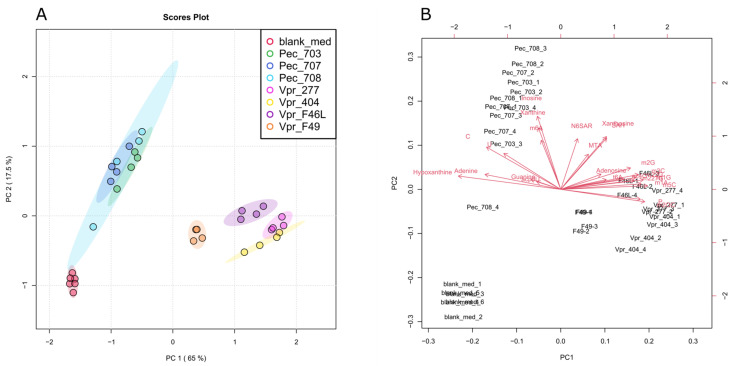
(**A**) Discrimination of investigated cell lines’ exometabolite profile by principal component analysis (PCA) indicating similarities and differences between samples and groups. Shaded area = confidence ellipses show 95% confidence regions. Percentage of variance explained by individual component is indicated, with PC 1 reaching 65 % variance and PC2 reaching 17.5% variance. Samples represented by dots. N = 4 for PEC and VPR, N = 6 for blank. (**B**) PCA Biplot displays how the individual metabolites contribute to the discrimination of the groups.

VPR cell lines derived from four independent VPR ccRCC tumors (277, 404, F46L and F49) secreted significantly higher amounts of modified nucleosides than cultures of primary renal epithelial cells (PECs) derived from three independent mice (703, 707 and 708). The results and statistical analyses are depicted in Supplementary Tables S1 and S2. In the PCA (principal component analysis), VPR cell lines, PEC cells and blank medium form clearly distinguishable groups (Figure 1A). PC 1 (principal component 1) has the biggest influence on the separation of the three groups, where the PEC and blank group lie closer together on PC 1 having the largest distance to the VPR group. PEC cells could be separated from the VPR cell lines and blank medium, with PC 1 reaching a variance of 65% and PC 2 reaching 17.5%, respectively. PC 2 has no effect on the separation between cancer and control. The Biplot, generated from the PCA (Figure 1B), highlights features, in this case nucleosides, which have a substantial influence on the separation of each group in a particular direction. A trend in which unmodified nucleosides such as C (cytidine), G (guanosine), I (inosine), A (adenosine), U (Uridine) having vectors pointing to the left and modified nucleosides such as m2OC (2′-O-methylcytidine), m3C (3-methylcytidine), m5C (5-methylcytidine), Ψ (pseudouridine), m1A (1-methyladenosine), m1G (1-methylguanosine), m227G (N^2^,N^2^,N^7^-trimethylguanosine), m22G (N^2^-N^2^-dimethylguanosine) and m2G (N^2^-methylguanosine), having vectors pointing to the right side, was observable. A feature’s vector parallel to the PC has a big impact on the separation of a sample in this direction. This implies that modified nucleosides cause this grouping on PC 1 in cancer and non-cancer. PC 2 also groups the samples, namely in used and unused medium, described by a feature’s vectors pointing upwards. These features are components of the medium, which were either consumed from or excreted into the medium. 

**Figure 2:**
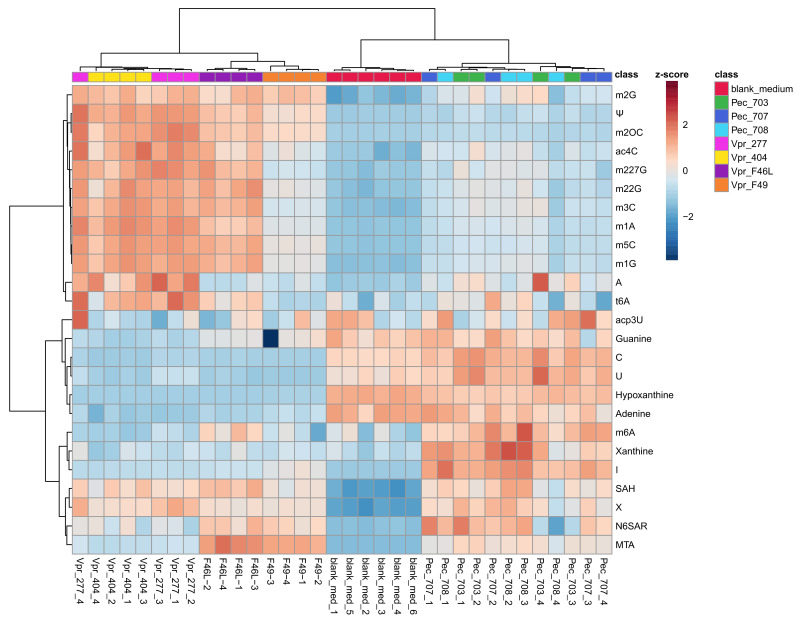
Heat map of the VPR, PEC cells and blank medium’s exometabolite profile. Rows refer to compounds and columns refer to biological samples. Three different PEC cell lines, four different VPR cell lines and the blank medium were analyzed. Only significantly altered metabolites (one-way ANOVA, corrected for multiple testing by FDR, *q*-value < 0.05) are displayed. The color scale on the right represents the range scaled z-score: redder tints refer to higher relative amounts and bluer tints refer to lower relative amounts of a respective metabolite in a biological sample. The clustering of the heat map demonstrates that the distances of the whole VPR group are greater than the distances between the PEC group and the blank control, which are clustered closer together. N = 4 for PEC and VPR, N = 6 for blank.

As depicted in the heat map (Figure 2), PEC cell lines and blanks were clustered close together, whereas VPR cell lines formed a separate cluster. This emphasizes the altered metabolism of VPR cell lines in contrast to PEC cell lines. It can be clearly seen that modified nucleosides were increased in VPR cell lines and were low in the PEC cells and blank medium. The relative concentrations of the respective metabolites are represented by the colors of the z-score. Higher relative concentrations are depicted in a redder tint, whereas lower concentrations are represented by more blue tints.

C was detected at comparable levels in the PEC cell medium and blank medium but was almost absent in the VPR cell culture medium. Thus, we propose that VPR cells take up C from the medium to build its derivatives m2OC, m1C, m3C, m5C and ac4C (N^4^-acetylcytidine). Similarly to C, the nucleobases hypoxanthine, guanine and adenine were present in lower amounts in the VPR medium than in the PEC medium, which means that these nucleobases were consumed for metabolism or even modified due to the higher growth and metabolism rates of cancer cells. 

The same applies for U, present in the blank and PEC medium, while its derivative Ψ was increased in the VPR medium. Indeed, U was almost entirely consumed by the VPR cells. U is converted to Ψ by Ψ-Synthases, making it more stable due to its C-glycosidic bond. U and Ψ were possibly both integrated in tRNAs of the VPR cells because of their increased metabolism and nucleic acid turnover due to their increased rate of growth. Ψ, being the most abundant modified nucleoside, is excreted into urine mainly as a degradation product of tRNA. In the past, Rasmuson et al. demonstrated that Ψ might serve as a prognostic marker for RCC, when they found Ψ correlated with tumor size and grade and the survival time was significantly decreased in patients with increased excretion [30].

Additionally, m1A, m1G, m227G, m22G and m2G were present in high concentrations in VPR cell lines shown in the heat map (Figure 2), depicted in reddish tints, while the same molecules were not present or only in low amounts in PEC cell lines and the blank medium. This coincides with former findings, where it has been demonstrated that modified nucleoside residues were increased in different cancer types such as prostate or breast [12,13]. The common nucleoside A was found to be elevated in some VPR cell lines and was increased in all cell media in which cells were grown. Extracellular A was found to have a suppressive effect on the activity of cytotoxic T cells against cancer cells causing immune suppression, which ultimately favors tumor progression and antitumor immunity [31]. Many physiological processes such as biosynthesis or regulations require methylations, which are realized by methyl transferases. Nucleosides are modified by methyl transferases from the NSUN family or METTLs (methyl transferase-like proteins) [32]. Several studies in the past have shown that the methyl transferase NSUN2 was overexpressed in different types of cancers such as breast, pancreatic, kidney or colorectal [33,34]. This circumstance might be reflected by the excreted methylated C species in VPR cells and is further confirmed by our transcriptomics analysis. The methyl group donor SAM (S-adenosylmethionine) leaves behind SAH (S-adenosylhomocysteine). We found SAH to be increased in both VPR and PEC cell lines. Methylations are a common reaction type in living cells, such as in DNA or RNA, for different reasons such as chromatin inactivation or epigenetic or transcriptomic purposes. Therefore, methylations of nucleosides do not seem to trigger an increase in extracellular SAH in a significant manner. 

**Figure 3:**
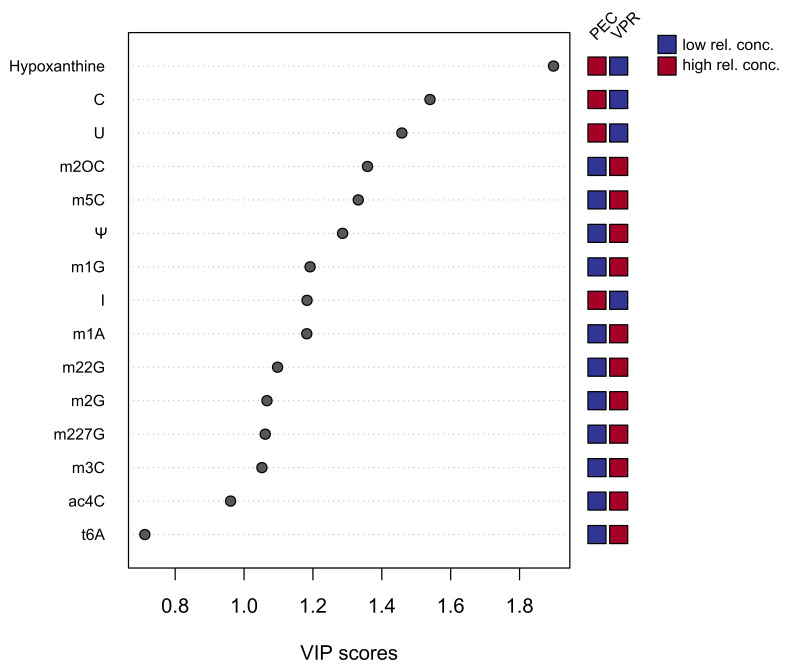
Most significant features of metabolites based on PLSDA-VIP score of component 1. Red and blue boxes indicate the relative concentrations of the respective nucleoside in each group of the study. (PEC = proximal tubular epithelial cells; VPR = triple knockout for *Vhl*, *Trp53* and *Rb1*). Nucleosides such as m2OC, m5C, m1G, m1A, m22G, m2G, m3C, Ψ and m227G have high VIP scores and therefore are the most contributory variables in the class discrimination of the PLSDA model, making them the most relevant features.

To analyze this dataset in a more robust way, we grouped cancer and non-cancer cells and conducted a PLSDA (partial-least squares discriminant analysis) and generated VIP scores (variable importance in projection), which are plotted in Figure 3. This scores plot shows the most significant features. The VIP score is a measure of a variable’s importance and features, with higher scores considered more relevant. It sums up which contribution a variable makes to the model. Features with VIP scores between 1.8 and 1.2, as well as features with scores between 0.8 and 1.2, are shown in Figure 3. Red and blue boxes refer to higher or lower relative concentrations, respectively. It was observed that modified nucleosides such as m2OC, m5C, m1G, m1A, m22G, m3C, Ψ and m227G were excreted in greater amounts into the VPR medium than in the PEC medium. This makes them the most contributory variables in the class discrimination of the PLSDA model. These findings emphasize that these compounds could have potential as biomarkers for ccRCC. 

### 3.2. Analysis under Low-Serum Conditions

The previous results were obtained in standard cell culture conditions. Since cancer cells in real tumor tissue are exposed to different nutritional conditions, we wanted to investigate whether it is possible to reproduce our results in starved medium conditions. For this analysis, we used a set of three primary cancer cell lines from the VPR model (104_RT, 306_RK and 332_LT), which had been generated independently from the previous experiment.

**Figure 4:**
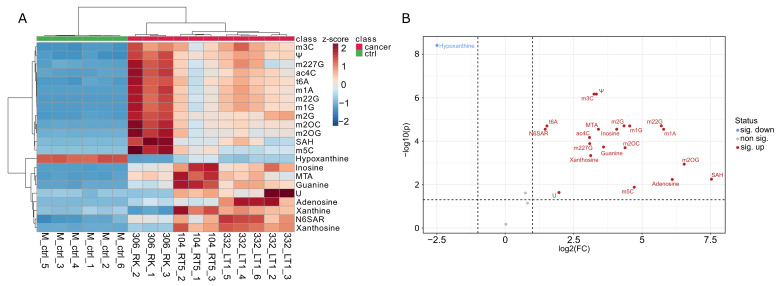
(**A**) Heat map analysis of the starvation approach. This analysis proves the previous findings. Modified residues were excreted into the medium. Hypoxanthine from the medium is consumed by the cells. The color scale on the right represents the relative concentrations of the respective nucleosides. (**B**) Volcano plot of the starvation approach. Significantly increased or decreased metabolites are displayed. Modified residues that were shown to be excreted in the rich medium are also increased in the starvation medium. The increase in the respective compound is represented by log_2_ of the fold change, with the y-axis representing the *p*-value.

The VPR cells grown under low-serum conditions (0.5% FCS) exhibited a comparable nucleoside profile to the VPR cells grown under 10% FCS conditions. Normalized nucleoside intensities and statistical results are shown in Supplementary Tables S3 and S4. These findings corroborate the method’s validity regarding its outcomes and stability. The LC-MS measurement of these samples demonstrated that the VPR cells excrete modified nucleosides in detectable amounts even under low-serum conditions comparable to the 10% serum condition. The corresponding heat map (Figure 4A) depicts the three measured VPR cell lines in triplicate and one cell line measured in a pentaplicate as well as six blank medium controls. For each sample, the respective nucleosides were analyzed, and it is clearly visible that modified nucleosides were excreted in the blank medium. The respective VPR cell lines were clustered in distinguishable groups, indicating their similarities to themselves and the differences to the control regarding their metabolism. One can also see that hypoxanthine from the medium was almost entirely consumed by the cells. The volcano plot (Figure 4B) proves this circumstance by displaying the significantly increased or decreased compounds. It can be seen that modified residues were excreted, and hypoxanthine from the medium was consumed. The volcano plot confirms our findings even under a nutrient-depleted environment. Importantly, Ψ, m3C, m5C and m2OC were among the substantially secreted nucleosides, as seen in the other, independent ccRCC cell lines. This highly reproducible feature of the VPR mouse model highlights them as promising candidates for ccRCC biomarkers.

### 3.3. Comparison of Results to Transcript Levels

**Figure 5:**
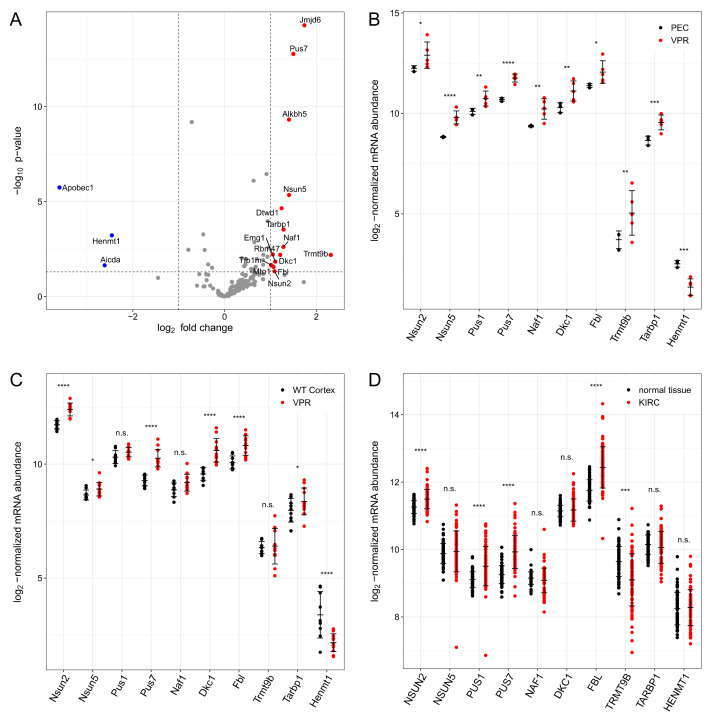
Analysis of RNA-modification genes. (**A**) The log_2_-fold changes (5 VPR cell lines compared to 3 PEC samples) and corresponding *p*-values of 152 genes involved in RNA modification are plotted. The 3 genes that decreased more than two-fold are depicted in blue and the 15 genes that increased more than two-fold are labeled in red. The dashed lines indicate the log_2_ fold change cut-offs (vertical lines at −1 and 1) and *p*-value cut-off (horizontal line at 0.05). Statistics were obtained using DESeq2. (**B**–**D**) The log_2_-normalized mRNA abundance is shown in the same VPR cell lines (N = 5) and PEC (N = 3) (**B**), in VPR tumors (N = 12) and WT cortex (N = 9) (**C**), and in human tumors (TCGA KIRC (Kidney Renal Clear Cell Carcinoma), N = 72) and matched normal tissue (N = 72) (**D**). Only those genes are shown which match the cut-offs and which produce the detected modified nucleosides. Error bars indicate the mean and standard deviation. The *p*-values were obtained from DESeq2 using Wald statistics (n.s. not significant, * *p* < 0.05, ** *p* < 0.01, *** *p* < 0.001, **** *p* < 0.0001).

To further validate our results with an orthogonal technique, we analyzed our previously described RNA-Seq dataset comparing VPR cells to PECs, followed by a generally applicable gene set enrichment (GAGE) analysis [26,35]. We focused on processes covering RNA modification (adjusted *p*-value 3.469157 × 10^−37^; unpaired non-parametric Kolmogorov–Smirnov test with FDR *q*-value adjustment using Benjamini–Hochberg correction), including methylation, aminoacylation and pseudouridine synthesis (Supplementary Table S5). We plotted the log_2_ fold change and corresponding *p*-values of genes in this GSEA (gene set enrichment analysis) term (Figure 5A). Out of 152 genes, 15 were upregulated more than two-fold, while 3 genes were decreased by a factor greater than two (Figure 5A). The significantly up-regulated genes encode enzymes, which produce nucleoside modifications detected in our LC-QqQ-MS experiments. *Trmt9b* and *Tarbp1* encode probable RNA-methyltransferases, *Nsun5* and *Nsun2* encode enzymes that produce m5C, of while *Pus7*, *Naf1*, *Dkc1* and *Pus1* (the latter was significantly increased by a factor 1.91) encode enzymes that are involved in Ψ synthesis [36,37,38,39]. *Fbl* encodes a 2′-O-methyltransferase [40]. On the other hand, *Henmt1* is reduced and encodes a 2′-O-methyltransferase specifically acting on piRNAs for their stabilization [41]. The up-regulation of the *Alkbh5* gene, an m6A demethylase in the PEC cells was also reflected in our metabolomics data, since m6A was significantly more abundant in the PEC medium. The role of Alkbh5 has been demonstrated in many biological processes such as metastasis formation, proliferation, invasion, migration and tumor growth [42]. Jmjd6 is a bifunctional arginine demethylase and lysyl–hydroxylase of histones and is possibly capable of binding single-stranded RNAs, where it might be able to modify nucleoside residues. Jmjd6 was upregulated in VPR cell lines, as seen in Figure 5A. In the literature, Jmjd6 was found to be elevated in several cancers such as prostate, lung, colon or breast cancer [43,44,45]. Dtwd1 is an enzyme involved in the synthesis of acp3U [46], and it was found to be increased in VPR cells. However, we could not detect alterations of acp3U in our setup, possibly due to the high U to Ψ turnover. Tfb1m is a SAM-dependent, mitochondrial adenosine dimethylase converting A to *N*^6^,*N*^6^-Dimethyladenosine [47], which was upregulated in VPR cells. This nucleoside modification was not detected in our LC-QqQ-MS method. *Apobec1* encodes an enzyme converting C post-transcriptionally to U in RNA [48]. We cannot elucidate whether a lower C to U conversion contributed to the extracellular nucleoside pool since both U and C were highly consumed for modification themselves. Although *Aicda* was significantly decreased in VPR cells, we did not focus on this enzyme as it acts exclusively on DNA [49]. RBM47 is an RNA-binding protein involved in many biological processes, as reviewed in [50]. EMG1 is an enzyme post-transcriptionally modifiying Ψ residues in rRNAs, converting them to the hyper modified N1-methyl-N3-(3-amino-3-carboxypropyl) pseudouridine (m1acp3Y) [51]. EMG1 was upregulated in VPR cells, but m1acp3Y was not included in our LC-QqQ-MS method. In further analyses, this modification should be included in the target list. Mto1 is a protein involved in the hyper modification of U in mitochondrial tRNAs to form 5-carboxymethylaminomethyl-2-thiouridine (cmnm5s2U) and was found to have increased expression in VPR cell lines [52]. 

**Figure 6:**
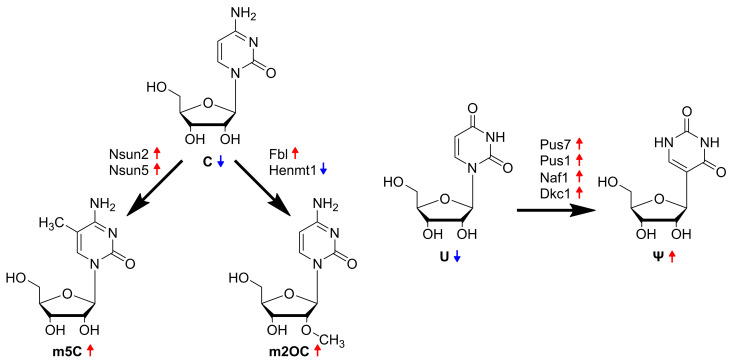
Summary of key findings: In ccRCC cells derived from the VPR mouse model, the modified nucleosides m5C, m2OC and Ψ, in addition to others, are excreted to the extracellular space. Accordingly, their unmodified precursor nucleosides C and U are reduced (indicated by a blue arrow pointing downwards). Several nucleoside-modifying enzymes responsible for these conversions are elevated in VPR cancer cells (indicated by a red arrow pointing upwards).

Thus, 9 out of the 15 regulated RNA modification genes encode proteins that could feasibly explain the elevated levels of the modified nucleosides m5C, m2OC and Ψ, highlighting them as putative biomarkers of ccRCC. Figure 5B shows selected genes responsible for the most contributory nucleoside modification in VPR ccRCC cells, as shown in Figure 3. Figure 6 summarizes the observed alterations in gene expression and nucleoside modifications in VPR ccRCC cells.

We next investigated the expression of selected RNA-modifying genes in our previously generated RNA-seq dataset comparing biopsies of VPR ccRCC tumors to biopsies of normal renal cortex to investigate whether the gene expression changes are also observed in vivo (Figure 5C) [9]. *Nsun2*, *Nsun5*, *Pus7*, *Dkc1*, *Fbl* and *Tarbp1* were all similarly upregulated in tumor tissue compared to normal, while *Henmt1* was similarly downregulated. Finally, we compared the expression of human homologs of these genes in ccRCC tumors from the TCGA KIRC study with patient-matched normal kidney biopsies (Figure 5D) [53,54,55,56]. *NSUN2*, *PUS1*, *PUS7* and *FBL* expression levels were statistically significantly higher in ccRCC tumor samples, while the other genes were unchanged. Keeping in mind that the bulk RNA-seq of tumor and tissue biopsies includes gene expression contributions from non-tumor and non-epithelial cells, respectively, these results argue that at least some aspects of the gene expression changes observed in our VPR cell culture models are also reflected in the in vivo setting in mouse and human ccRCC tumors.

## 4. Conclusions

To the best of our knowledge, this is the first study of modified nucleosides in a ccRCC cell model. We were able to demonstrate that VPR cells secrete significant amounts of modified nucleoside residues in contrast to PEC control cells. The most relevant compounds we identified were derivatives of common nucleosides such as C, G, A and U. One can also deduce that VPR cell lines take up common nucleosides from the medium to fuel their rapid metabolism, using them for growth and modifying them, ultimately making them not salvageable, which causes their excretion into the cell culture medium. We demonstrated that cancer-related nucleosides, which were previously proven to be clinically relevant in cancers, were also increased in our cell model for ccRCC. We successfully established a method for the analysis of modified nucleosides in ccRCC by LC-QqQ-MS, which makes us believe that this method is applicable for other biological samples such as plasma, serum and urine. Gene expression analyses identified the upregulation of a series of genes that regulate nucleoside modification in ccRCC cells and mouse and human tumors, providing a plausible mechanism for the observed metabolic observations and providing a basis for future genetic interventional studies to modify the expression of these genes and characterize the effects on nucleoside metabolism and cellular proliferation. Finally, our findings identified potential candidate biomarkers for ccRCC, which provides the basis for further clinical studies to determine whether modified nucleosides might represent a diagnostic tool that could be applied in the context of ccRCC detection and monitoring. Therefore, our mass-spectrometry-based metabolomics approach for the biomarker analysis of ccRCC seems to be promising in, e.g., urinary samples, since modified nucleosides excreted by tumor cells ultimately accumulate in urine. Finally, a metabolic nucleoside pattern, combined with genomics, transcriptomics or proteomics data which are specific for ccRCC, might be used as a robust biomarker. More specifically, we suggest that the U to Ψ, C to m5C and C to m2OC ratios, as well as the combination of the significantly altered nucleosides in combination with their corresponding enzyme mRNA levels, could be used as potential biomarkers, which should be validated in further clinical trials.

## Figures and Tables

**Table 1:** Analyzed nucleosides/nucleobases and their abbreviations.

Nucleoside/Nucleobase	Abbreviation
N^4^-Acetylcytidine	ac4C
3-(3-Amino-carboxypropyl)-uridine	acp3U
Adenine	Adenine
Adenosine	A
Cytidine	C
Guanine	Guanine
Guanosine	G
Hypoxanthine	Hypoxanthine
Inosine	I
1-Methyladenosine	m1A
1-Methylguanosine	m1G
N^2^,N^2^,N^7^-Trimethylguanosine	m227G
N^2^-N^2^-Dimethylguanosine	m22G
N^2^-Methylguanosine	m2G
2′-O-Methylcytidin	m2OC
2′-O-Methylguanosine	m2OG
3-Methylcytidine	m3C
5-Methylcytidine	m5C
6-Methyladenosine	m6A
5′-Deoxy-5′-Methylthioadenosine	MTA
N^6^-Succinyloadenosine	N6SAR
Pseudouridine	Ψ
S-Adenosylhomocysteine	SAH
N^6^-Threonyl-carbamoyladenosine	t6A
Uridine	U
Xanthine	Xanthine
Xanthosine	X

## Data Availability

Results are shown in Supplementary Tables S1 and S3. Results of statistical analyses are shown in Supplementary Tables S2 and S4. RNA data are shown in Supplementary Table S5.

## Supplementary Materials

The following supporting information can be downloaded at: https://www.mdpi.com/article/10.3390/cells12081102/s1: Table S1: Intensity table of nucleosides detected in rich growth medium; Table S2: Intensity table of nucleosides detected in medium with 0.5% serum; Table S3: Statistical results of nucleosides from rich growth medium; Table S4: Statistical results of nucleosides from medium containing 0.5% serum; Table S5: RNA: expression data.

## References

[1] Sung H., Ferlay J., Siegel R.L., Laversanne M., Soerjomataram I., Jemal A., Bray F. (2021). Global Cancer Statistics 2020: GLOBOCAN Estimates of Incidence and Mortality Worldwide for 36 Cancers in 185 Countries. CA Cancer J. Clin..

[2] Hsieh J.J., Purdue M.P., Signoretti S., Swanton C., Albiges L., Schmidinger M., Heng D.Y., Larkin J., Ficarra V. (2017). Renal cell carcinoma. Nat. Rev. Dis. Prim..

[3] Li Q.K., Pavlovich C.P., Zhang H., Kinsinger C.R., Chan D.W. (2019). Challenges and opportunities in the proteomic characterization of clear cell renal cell carcinoma (ccRCC): A critical step towards the personalized care of renal cancers. Semin. Cancer Biol..

[4] Frew I.J., Moch H. (2015). A clearer view of the molecular complexity of clear cell renal cell carcinoma. Annu. Rev. Pathol..

[5] Sato Y., Yoshizato T., Shiraishi Y., Maekawa S., Okuno Y., Kamura T., Shimamura T., Sato-Otsubo A., Nagae G., Suzuki H. (2013). Integrated molecular analysis of clear-cell renal cell carcinoma. Nat. Genet..

[6] (2013). Comprehensive molecular characterization of clear cell renal cell carcinoma. Nature.

[7] Semenza G.L. (2013). HIF-1 mediates metabolic responses to intratumoral hypoxia and oncogenic mutations. J. Clin. Investig..

[8] Masson N., Ratcliffe P.J. (2014). Hypoxia signaling pathways in cancer metabolism: The importance of co-selecting interconnected physiological pathways. Cancer Metab..

[9] Hoefflin R., Harlander S., Schäfer S., Metzger P., Kuo F., Schönenberger D., Adlesic M., Peighambari A., Seidel P., Chen C.-Y. (2020). HIF-1α and HIF-2α differently regulate tumour development and inflammation of clear cell renal cell carcinoma in mice. Nat. Commun..

[10] Reuter V.E., Tickoo S.K. (2010). Differential diagnosis of renal tumours with clear cell histology. Pathology.

[11] Hakimi A.A., Reznik E., Lee C.-H., Creighton C.J., Brannon A.R., Luna A., Aksoy B.A., Liu E.M., Shen R., Lee W. (2016). An Integrated Metabolic Atlas of Clear Cell Renal Cell Carcinoma. Cancer Cell.

[12] Willmann L., Erbes T., Halbach S., Brummer T., Jäger M., Hirschfeld M., Fehm T., Neubauer H., Stickeler E., Kammerer B. (2015). Exometabolom analysis of breast cancer cell lines: Metabolic signature. Sci. Rep..

[13] Lagies S., Schlimpert M., Braun L.M., Kather M., Plagge J., Erbes T., Wittel U.A., Kammerer B. (2019). Unraveling altered RNA metabolism in pancreatic cancer cells by liquid-chromatography coupling to ion mobility mass spectrometry. Anal. Bioanal. Chem..

[14] Frickenschmidt A., Frohlich H., Bullinger D., Zell A., Laufer S., Gleiter C.H., Liebich H., Kammerer B. (2008). Metabonomics in cancer diagnosis: Mass spectrometry-based profiling of urinary nucleosides from breast cancer patients. Biomark. Biochem. Indic. Expo. Response Susceptibility Chem..

[15] Cantara W.A., Crain P.F., Rozenski J., McCloskey J.A., Harris K.A., Zhang X., Vendeix F.A.P., Fabris D., Agris P.F. (2011). The RNA Modification Database, RNAMDB: 2011 update. Nucleic Acids Res..

[16] Boschi-Muller S., Motorin Y. (2013). Chemistry enters nucleic acids biology: Enzymatic mechanisms of RNA modification. Biochemistry.

[17] Boccaletto P., Stefaniak F., Ray A., Cappannini A., Mukherjee S., Purta E., Kurkowska M., Shirvanizadeh N., Destefanis E., Groza P. (2022). MODOMICS: A database of RNA modification pathways. 2021 update. Nucleic Acids Res..

[18] Limbach P.A., Crain P.F., McCloskey J.A. (1994). Summary: The modified nucleosides of RNA. Nucleic Acids Res..

[19] Sloan K.E., Warda A.S., Sharma S., Entian K.-D., Lafontaine D.L.J., Bohnsack M.T. (2017). Tuning the ribosome: The influence of rRNA modification on eukaryotic ribosome biogenesis and function. RNA Biol..

[20] Willmann L., Erbes T., Krieger S., Trafkowski J., Rodamer M., Kammerer B. (2015). Metabolome analysis via comprehensive two-dimensional liquid chromatography: Identification of modified nucleosides from RNA metabolism. Anal. Bioanal. Chem..

[21] Kammerer B., Frickenschmidt A., Gleiter C.H., Laufer S., Liebich H. (2005). MALDI-TOF MS analysis of urinary nucleosides. J. Am. Soc. Mass Spectrom..

[22] Kammerer B., Frickenschmidt A., Müller C.E., Laufer S., Gleiter C.H., Liebich H. (2005). Mass spectrometric identification of modified urinary nucleosides used as potential biomedical markers by LC-ITMS coupling. Anal. Bioanal. Chem..

[23] Bullinger D., Fröhlich H., Klaus F., Neubauer H., Frickenschmidt A., Henneges C., Zell A., Laufer S., Gleiter C.H., Liebich H. (2008). Bioinformatical evaluation of modified nucleosides as biomedical markers in diagnosis of breast cancer. Anal. Chim. Acta.

[24] Bullinger D., Neubauer H., Fehm T., Laufer S., Gleiter C.H., Kammerer B. (2007). Metabolic signature of breast cancer cell line MCF-7: Profiling of modified nucleosides via LC-IT MS coupling. BMC Biochem..

[25] Harlander S., Schönenberger D., Toussaint N.C., Prummer M., Catalano A., Brandt L., Moch H., Wild P.J., Frew I.J. (2017). Combined mutation in Vhl, Trp53 and Rb1 causes clear cell renal cell carcinoma in mice. Nat. Med..

[26] Seidel P., Rubarth A., Zodel K., Peighambari A., Neumann F., Federkiel Y., Huang H., Hoefflin R., Adlesic M., Witt C. (2022). ATR represents a therapeutic vulnerability in clear cell renal cell carcinoma. JCI Insight.

[27] Schlimpert M., Lagies S., Budnyk V., Müller B., Walz G., Kammerer B. (2018). Metabolic Phenotyping of Anks3 Depletion in mIMCD-3 cells—a Putative Nephronophthisis Candidate. Sci. Rep..

[28] Xia J., Psychogios N., Young N., Wishart D.S. (2009). MetaboAnalyst: A web server for metabolomic data analysis and interpretation. Nucleic Acids Res..

[29] Van den Berg R.A., Hoefsloot H.C.J., Westerhuis J.A., Smilde A.K., van der Werf M.J. (2006). Centering, scaling, and transformations: Improving the biological information content of metabolomics data. BMC Genom..

[30] Rasmuson T., Björk G.R., Hietala S.O., Stenling R., Ljungberg B. (1991). Excretion of pseudouridine as an independent prognostic factor in renal cell carcinoma. Acta Oncol..

[31] Allard B., Allard D., Buisseret L., Stagg J. (2020). The adenosine pathway in immuno-oncology. Nat. Rev. Clin. Oncol..

[32] Xu L., Liu X., Sheng N., Oo K.S., Liang J., Chionh Y.H., Xu J., Ye F., Gao Y.-G., Dedon P.C. (2017). Three distinct 3-methylcytidine (m3C) methyltransferases modify tRNA and mRNA in mice and humans. J. Biol. Chem..

[33] Chellamuthu A., Gray S.G. (2020). The RNA Methyltransferase NSUN2 and Its Potential Roles in Cancer. Cells.

[34] Okamoto M., Hirata S., Sato S., Koga S., Fujii M., Qi G., Ogawa I., Takata T., Shimamoto F., Tatsuka M. (2012). Frequent increased gene copy number and high protein expression of tRNA (cytosine-5-)-methyltransferase (NSUN2) in human cancers. DNA Cell Biol..

[35] Luo W., Friedman M.S., Shedden K., Hankenson K.D., Woolf P.J. (2009). GAGE: Generally applicable gene set enrichment for pathway analysis. BMC Bioinform..

[36] The UniProt Consortium (2023). UniProt: The Universal Protein Knowledgebase in 2023. Nucleic Acids Res..

[37] Bohnsack K.E., Höbartner C., Bohnsack M.T. (2019). Eukaryotic 5-methylcytosine (m⁵C) RNA Methyltransferases: Mechanisms, Cellular Functions, and Links to Disease. Genes.

[38] Cerneckis J., Cui Q., He C., Yi C., Shi Y. (2022). Decoding pseudouridine: An emerging target for therapeutic development. Trends Pharmacol. Sci..

[39] Kiss T., Fayet-Lebaron E., Jády B.E. (2010). Box H/ACA small ribonucleoproteins. Mol. Cell.

[40] Shubina M.Y., Musinova Y.R., Sheval E.V. (2016). Nucleolar methyltransferase fibrillarin: Evolution of structure and functions. Biochemistry.

[41] Lim S.L., Qu Z.P., Kortschak R.D., Lawrence D.M., Geoghegan J., Hempfling A.-L., Bergmann M., Goodnow C.C., Ormandy C.J., Wong L. (2015). HENMT1 and piRNA Stability Are Required for Adult Male Germ Cell Transposon Repression and to Define the Spermatogenic Program in the Mouse. PLoS Genet..

[42] Wang J., Wang J., Gu Q., Ma Y., Yang Y., Zhu J., Zhang Q.'A. (2020). The biological function of m6A demethylase ALKBH5 and its role in human disease. Cancer Cell Int..

[43] Hong X., Zang J., White J., Wang C., Pan C.-H., Zhao R., Murphy R.C., Dai S., Henson P., Kappler J.W. (2010). Interaction of JMJD6 with single-stranded RNA. Proc. Natl. Acad. Sci. USA.

[44] Konuma T., Yu D., Zhao C., Ju Y., Sharma R., Ren C., Zhang Q., Zhou M.-M., Zeng L. (2017). Structural Mechanism of the Oxygenase JMJD6 Recognition by the Extraterminal (ET) Domain of BRD4. Sci. Rep..

[45] Wang K., Yang C., Li H., Liu X., Zheng M., Xuan Z., Mei Z., Wang H. (2022). Role of the Epigenetic Modifier JMJD6 in Tumor Development and Regulation of Immune Response. Front. Immunol..

[46] Takakura M., Ishiguro K., Akichika S., Miyauchi K., Suzuki T. (2019). Biogenesis and functions of aminocarboxypropyluridine in tRNA. Nat. Commun..

[47] Metodiev M.D., Lesko N., Park C.B., Cámara Y., Shi Y., Wibom R., Hultenby K., Gustafsson C.M., Larsson N.-G. (2009). Methylation of 12S rRNA is necessary for in vivo stability of the small subunit of the mammalian mitochondrial ribosome. Cell Metab..

[48] Blanc V., Davidson N.O. (2010). APOBEC-1-mediated RNA editing. Wiley Interdiscip. Rev. Syst. Biol. Med..

[49] Rios L.A.D.S., Cloete B., Mowla S. (2020). Activation-induced cytidine deaminase: In sickness and in health. J. Cancer Res. Clin. Oncol..

[50] Shivalingappa P.K.M., Sharma V., Shiras A., Bapat S.A. (2021). RNA binding motif 47 (RBM47): Emerging roles in vertebrate development, RNA editing and cancer. Mol. Cell Biochem..

[51] Wurm J.P., Meyer B., Bahr U., Held M., Frolow O., Kötter P., Engels J.W., Heckel A., Karas M., Entian K.-D. (2010). The ribosome assembly factor Nep1 responsible for Bowen-Conradi syndrome is a pseudouridine-N1-specific methyltransferase. Nucleic Acids Res..

[52] Li X., Li R., Lin X., Guan M.-X. (2002). Isolation and characterization of the putative nuclear modifier gene MTO1 involved in the pathogenesis of deafness-associated mitochondrial 12 S rRNA A1555G mutation. J. Biol. Chem..

[53] Clark K., Vendt B., Smith K., Freymann J., Kirby J., Koppel P., Moore S., Phillips S., Maffitt D., Pringle M. (2013). The Cancer Imaging Archive (TCIA): Maintaining and operating a public information repository. J. Digit. Imaging.

[54] Akin O., Elnajjar P., Heller M., Jarosz R., Erickson B.J., Kirk S., Lee Y., Linehan M.W., Gautam R., Vikram R. (2016). The Cancer Genome Atlas Kidney Renal Clear Cell Carcinoma Collection (TCGA-KIRC).

[55] Colaprico A., Silva T.C., Olsen C., Garofano L., Cava C., Garolini D., Sabedot T.S., Malta T.M., Pagnotta S.M., Castiglioni I. (2016). TCGAbiolinks: An R/Bioconductor package for integrative analysis of TCGA data. Nucleic Acids Res..

[56] Grossman R.L., Heath A.P., Ferretti V., Varmus H.E., Lowy D.R., Kibbe W.A., Staudt L.M. (2016). Toward a Shared Vision for Cancer Genomic Data. N. Engl. J. Med..

